# Supplementary material for: Interpretable ensemble machine learning framework for cardiovascular disease prediction using EMR data and large language models in Ethiopia
Source: PLoS One. 2026 Feb 9;21(2):e0342256. doi: 10.1371/journal.pone.0342256 (PMC12885298; doi:10.1371/journal.pone.0342256)
Supplement: Attachment — Submitted filename: Response to Reviewers.docx [file pone.0342256.s001.docx]

| **No** | **Comments** | **Author Response** | **Justification / Revision Action** |
| --- | --- | --- | --- |
| **1** | **Is the manuscript technically sound, and do the data support the conclusions?**  The manuscript must describe a technically sound piece of scientific research with data that supports the conclusions. Experiments must have been conducted rigorously, with appropriate controls, replication, and sample sizes. The conclusions must be drawn appropriately based on the data presented.**Reviewer #1:** No**Reviewer #2:** Partly | We thank the reviewers for their feedback. The revised manuscript now provides a clearer and more detailed explanation of the predictive modeling process. Specifically, Sections 3.2 and 3.3 have been expanded to include all preprocessing, feature handling, and parameter settings for the XGBoost model. Additionally, we clarified how the results align with the stated objectives in both the Discussion and Conclusion sections. | The predictive pipeline now explicitly covers missing value handling, outlier treatment, feature encoding, dimensionality reduction, and evaluation metrics. Tables 1–3 summarize dataset characteristics. The objective, method, and results are now clearly aligned in Section 5 to demonstrate methodological soundness and data-driven conclusions. |
| **2** | **Has the statistical analysis been performed appropriately and rigorously?Reviewer #1:** No**Reviewer #2:** No | We have strengthened the statistical analysis section to ensure transparency and rigor. The manuscript now clarifies that model validation was conducted using 10-fold cross-validation to ensure reliability and reduce overfitting. | Added detailed explanation in Section 3.3 on cross-validation and evaluation metrics (accuracy, precision, recall, F1-score). Figures 1(a–b) demonstrate convergence stability, confirming model robustness. |
| **3** | Have the authors made all data underlying the findings in their manuscript fully available? Reviewer #1: No Reviewer #2: Yes | The Data Availability Statement has been revised to comply fully with PLOS policy. It now states that anonymized data may be obtained at 10.6084/m9.figshare.30436426 | Revised statement under *Data Availability* section: explicitly clarifies data privacy restrictions, ethical approval, and data access procedures consistent with institutional and national regulations. |
| **4** | **Is the manuscript presented in an intelligible fashion and written in standard English?Reviewer #1:** Yes**Reviewer #2:** Yes | We appreciate the reviewers’ positive remarks regarding the clarity of the manuscript. The entire text has nevertheless been re-edited for grammar, readability, and logical flow to further enhance comprehension. | Revised by improving sentence structure and section transitions, particularly in Introduction, Related Works, and Methods sections. Minor typographical and grammatical issues have been corrected. |
| **5** | **Review Comments to the AuthorReviewer #1:** There is a huge variance between the results and objectives. The prediction method is omitted. Dataset description and selected features are unclear.**Reviewer #2:** Suggested exploring other ML/DL methods, model validation, restructuring introduction, clarifying methodology (data-driven term, CVD definition, variable selection, missingness, IQR formula, dimensionality reduction, standardization), inclusion/exclusion criteria, and improved flow. | **We are grateful for these comprehensive comments.** All major methodological and structural issues have been addressed as follows:  • **Prediction Method:** Section 3.3 fully describes the XGBoost model, hyperparameters, and validation strategy.  • **Feature Description:** Tables 1–3 and Section 3.2 detail preprocessing, feature types, and data transformations.  • **CVD Definition:** CVD is now defined as “presence of a documented cardiovascular diagnosis in the EMR (coded as 1) and absence as 0.”  • **Missing Data:** A new paragraph reports missingness percentages and clarifies that median imputation was applied only to continuous variables. Future work will employ multiple imputation.  • **IQR Formula:** Corrected to Q1 – 1.5×IQR and Q3 + 1.5×IQR  .• **Dimensionality Reduction:** Justification added—reduces noise, computational load, and overfitting.  • **Standardization:** Explained as necessary to normalize continuous clinical variables (pulse, respiratory rate, etc.).  • **Model Comparison:** Section 5 discusses why XGBoost was prioritized and mentions future testing with Random Forest, SVM, and DNN  .• **External Validation:** Added as a limitation; planned for future studies using independent hospital datasets.  • **Methods Clarification:** Replaced “experimental, data-driven design” with “retrospective, data-driven design using anonymized EMR records.”  • **Introduction & Related Works:** Restructured and integrated for improved readability and justification of research gap.  • **Sample Size & Inclusion Criteria:** Clarified that 20,960 anonymized EMR records were obtained on 23/02/2025, including patients with complete demographic and diagnosis fields. | The revised manuscript provides comprehensive methodological transparency:– Clear variable definitions, rationale, and citations added. Refer 3.1-3.5  – Section 3.1 details dataset source and collection date.  – Ethical and data confidentiality considerations clarified.  – Discussion now links objectives, findings, and implications  .– Future work highlights inclusion of multiple models and external validation.These revisions address all technical, structural, and conceptual concerns raised by both reviewers, ensuring methodological soundness and readability. |
