# Supplementary material for: Interpretable ensemble machine learning framework for cardiovascular disease prediction using EMR data and large language models in Ethiopia
Source: PLoS One. 2026 Feb 9;21(2):e0342256. doi: 10.1371/journal.pone.0342256 (PMC12885298; doi:10.1371/journal.pone.0342256)
Supplement: Attachment — Submitted filename: Response_to_Reviewers_auresp_2.docx [file pone.0342256.s002.docx]

Alemu Kumilachew Tegegnie

Bahir Dar University Instituite of Technology

December 18, 2025

To:

The Editor

PLOS ONE

**Subject:** Response to Reviewer Comments for Manuscript: *"Interpretable Ensemble Machine Learning Framework for Cardiovascular Disease Prediction Using EMR Data and Large Language Models in Ethiopia"*

Dear Editor and Reviewers,

We wish to express our sincere gratitude to the reviewers for their insightful and constructive feedback on our manuscript. Their comments have been instrumental in improving the statistical rigor and clinical transparency of this work.

Following the suggestions, we have revised the manuscript to clarify our variable definitions, justify our imputation strategy, and incorporate preliminary statistical analyses. We also updated the reference list to include citations for Ethiopian Primary Health Care Clinical Guidelines (2021) and EMR standard protocols and usage. Below is our point-by-point response to the reviewer’s comments.

**Response to Reviewer #2**

1. Comment on Variable Definition (CVD and Clinical Systems):

The author did not adequately explain how CVD was defined in this study... clearly articulate whether this was based on ICD code or self-reporting. The same applies to all clinical variables (Musculoskeletal, neurological, respiratory system)...

Response:

We agree that transparency regarding data sources is vital. We have updated Section 3.2 (Data Acquisition and Clinical Definitions) to clarify that all diagnoses, including CVD, were established by attending physicians using ICD-10 standards within the EMR, rather than self-reporting. Furthermore, clinical system variables were derived from standardized physical examination modules following the Ethiopian Primary Health Care Clinical Guidelines (2021).

**Revision in Manuscript (Section 3.2):**

"In this study, Cardiovascular Disease (CVD) was defined based on formal clinician diagnoses documented within the EMR system, utilizing ICD-10 standards. Clinical predictors such as the Musculoskeletal System (MSS) and Respiratory System (RESP_SYST) represent assessment modules within the EMR that capture findings from the 'Review of Systems' and physical examinations. These follow the Ethiopian Primary Health Care Clinical Guidelines (2021) and national EMR clinical documentation protocols."

2. Comment on Imputation Strategy:

Median imputation can underestimate variability and potentially bias the estimated associations... I recommend applying an imputation strategy (Like multiple imputation) for the outcome variable.

Response:

We appreciate this statistical insight. We have acknowledged the limitations of median imputation in Section 3.4 (Data Preprocessing and Imputation). While median imputation was initially chosen for its robustness against outliers in the EMR physiological data, we have now included a discussion on why binary variables were treated as distinct "Not Recorded" categories to avoid synthetic bias. We have also added a recommendation to utilize Multiple Imputation (MI) in future work to further validate the stability of these associations.

3. Comment on Bivariate Analysis:

The statistical analysis would benefit from additional rigor. I recommend that the authors include at least a bivariate analysis between the outcome variable and exposure variables or predictors before applying the ML methods.

Response:

We have integrated a new section, Section 3.5 (Preliminary Statistical Analysis and Feature Selection), which details the bivariate analysis conducted prior to machine learning. This includes the use of Pearson Chi-square (*X^2^*) tests for categorical variables and independent t-tests for continuous variables.

**Revision in Manuscript (Combined Section 3.5):**

"Before implementing the ensemble machine learning framework, a rigorous preliminary analysis was conducted to examine the data structure and the associations between predictors and the CVD outcome. Bivariate analysis was performed to evaluate these relationships: categorical variables (e.g., HPI, General Plan) were assessed using the Pearson Chi-square (*X*^2^)test, while continuous variables were compared using independent t-tests, depending on the normality of their distribution... This integrated approach ensures that the underlying clinical distribution is transparent and that the feature importance subsequently identified by the XGBoost and SHAP models is both statistically robust and clinically grounded."

We believe these revisions address all the concerns raised. We remain at your disposal for any further clarifications.

Sincerely,

Alemu Kumilachew Tegegnie & Kibrom Tewolde Siyum

Corresponding Authors
